# Women's preferences for integrating multi-product pre-exposure prophylaxis delivery programs within services for sexually transmitted infections and reproductive health care in Uganda: a discrete choice experiment

**DOI:** 10.3389/frph.2025.1688969

**Published:** 2025-11-10

**Authors:** Brenda Kamusiime, Patricia M. Smith, Alisaati Nalumansi, Tara E. Wood, George Eram, Vicent Kasiita, Paul Ssendiwala, Agnes Nakyanzi, Felix Bambia, Timothy R. Muwonge, Andrew Mujugira, Elizabeth T. Montgomery, Renee Heffron

**Affiliations:** 1 Infectious Diseases Institute, Makerere University, Kampala, Uganda; 2 Department of Medicine, University of Alabama at Birmingham, Birmingham, AL, United States; 3 Department of Global Health, University of Washington, Seattle, WA, United States; 4 Women’s Global Health Imperative, RTI International, Berkeley, CA, United States; 5 Department of Epidemiology and Biostatistics, University of California, San Francisco, CA, United States

**Keywords:** HIV, pre exposure prophylaxis (PrEP), discrete choice experiment (DCE), Uganda, adolescent girls and young women (AGYW), reproductive health (RH)

## Abstract

**Introduction:**

HIV prevention is paramount for adolescent girls and young women (AGYW) in Uganda, and oral pre-exposure prophylaxis (PrEP) is not always a suitable option. With emerging novel HIV prevention products (e.g., ring, injectables), there are opportunities to explore AGYW preferences to inform strategies for integrating PrEP choice into routine care.

**Methods:**

From January–September 2024, we recruited AGYW aged 16–25 years from community sites in Kampala, Uganda for a cross-sectional discrete choice experiment (DCE) to determine the most preferred attributes and levels of multi-product PrEP programs. The DCE was developed via literature review, informal conversations with AGYW, and cognitive interviewing among AGYW using a prototype instrument. In the final iteration, attributes (and levels) included: method of PrEP information dissemination (WhatsApp, brochure, in-person consultations), PrEP counseling delivery (virtual, group, in-person counselling), proximity of PrEP location (nearer to or far from work/school/home), type of facility (private or government clinic, pharmacy), availability of additional services (STI testing and treatment, family planning, no additional services), client wait times (5, 30, 90 min), and associated costs (small, none). Participants responded 9 times to the question “Which PrEP program would encourage you to use PrEP?” and each time a different set of randomly-assigned choices of 2 scenarios were presented. Multinomial logit modeling was used to estimate preference weights and importance scores.

**Results:**

Of 343 AGYW screened, 300 consented to participate (median age: 21 years, IQR: 20–23), with 38.3% having oral PrEP experience and 71.7% reporting recent condomless sex. “Access to other services” in conjunction with PrEP dispensing had the greatest influence on PrEP program choice (importance score: 27%) with preferences for STI testing and treatment (preference weight: 0.39, 95% CI: 0.32, 0.47) and family planning (PW: 0.14, 95% CI: 0.07, 0.21) greater than stand-alone PrEP programs. The type of facility offering PrEP (importance score: 9.7%), method used for PrEP information dissemination (importance score: 10.2%), and proximity of the PrEP location (importance score: 6.9%) were not very influential.

**Discussion:**

Young women's preference for PrEP services to be offered in conjunction with STI and/or reproductive health services indicates an opportunity to integrate current and future PrEP delivery within these existing services.

## Introduction

The 2025 Global AIDS Update reported a 56% reduction in the number of new HIV acquisitions in eastern and southern Africa (1). However despite the overall decrease, AGYW accounted for 28% of new HIV infections in the region, a disproportionate burden and three-fold increase compared to their male counterparts (2). Daily oral HIV pre-exposure prophylaxis (PrEP) has high efficacy and is an approved prevention strategy (3). Demonstration projects and data from programmatic delivery show high oral PrEP uptake among AGYW in eastern and southern Africa, yet many discontinue by 12 months or earlier, due in large part to a challenging daily dosing schedule. Side effects, frequent refill schedules, negative judgment by healthcare providers, and stigma are also cited as reasons influencing early discontinuation (4). Novel PrEP products, both approved and in roll-out phases regionally and in Uganda, include a 2-monthly injection with cabotegravir and a monthly dapivirine-eluting vaginal ring (5). More recently, a 6-monthly injection with lenacapavir was approved for use for HIV prevention, and other promising long-acting formulations are in development (6–9). The promise of novel PrEP products offers opportunities to understand AGYW experiences with daily oral PrEP and identify new or revamped strategies that meet their preferences and will facilitate upcoming multi-product PrEP programs to achieve high prevention coverage.

Currently in Uganda, oral PrEP is offered at government clinics, hospitals, HIV testing and counselling centers, implementation research and demonstration sites, and not-for-profit organizations (10–12). The dapivirine ring and 2-monthly injectable cabotegravir are included in Uganda HIV prevention clinical guidelines, and limited quantities were distributed in the country during a now-terminated USAID-supported demonstration project at 7 public health facilities (10). To reduce financial and programmatic dependence on foreign assistance, the Uganda Ministry of Health is working to restart delivery of these commodities, donated from multinational organizations. Originally rolled out in a phased manner in Uganda, oral PrEP was first offered to HIV serodifferent couples and later expanded to other key populations including men who have sex with men (MSM), sex workers, people who inject drugs, fisherfolk, transgender populations, adolescent girls and young women, and truck drivers (13). Newly introduced PrEP products could be integrated into existing oral PrEP programs, resulting in multi-product PrEP service delivery models. In line with this, the Uganda Ministry of Health is strongly recommending the integration of PrEP into routine clinical services, with existing clinic staff leading the effort to integrate HIV services within other health services; the goal being to shift away from stand-alone HIV and PrEP programs. To contribute data to inform future PrEP programmatic decision making, we conducted a discrete choice experiment (DCE) among AGYW, including those experienced with oral PrEP and PrEP-naïve.

## Materials and methods

We conducted a DCE among AGYW between the ages of 16 and 25 years, a population considered important candidates for PrEP, based on their sexual behaviour. Potential participants were recruited in Kampala, Uganda from PrEP studies previously conducted at the IDI-Kasangati research clinic and through partnerships with family planning centers, vocational training schools, and public clinics where both antiretroviral therapy and PrEP are dispensed. To gain rapport with clinics and schools, the study community liaison officer sensitised clinic/school staff and provided them with information about the study. All potential participants were referred to the study recruitment team and those meeting eligibility criteria were consecutively enrolled. Based on recommendations for discrete choice experiments and the number of attributes and levels in our DCE, a target enrollment goal of 300 participants was set (14, 15).

The study was conducted at Makerere University College of Health Sciences Infectious Diseases Institute's Kasangati clinical research site in Kampala, Uganda, from January to September 2024. Potential participants were invited to the research site and underwent testing for HIV according to national guidelines. Women meeting eligibility criteria who were willing and able to undergo informed consent were enrolled. Eligibility criteria for those ages 16–17 years included qualifying as an emancipated minor, according to Ugandan ethics regulations. On the same day as study screening, eligible AGYW who were willing to participate in the study underwent an informed consent process and completed the interviewer-administered questionnaires to capture their current and recent choices about PrEP use and their sexual behaviour, as well as the choice set questions comprising the DCE.

Prior to study launch, we used a two-step process to develop the DCE, including selecting attributes and defining levels for each attribute. Formative research, including literature reviews and informal interviews with PrEP-experienced AGYW, was used to develop initial attributes and levels. With these lists, we created a prototype of the DCE data collection instrument and then conducted six cognitive interviews where DCE language and pictorial representations of attribute levels were discussed with AGYW. Thorough review by the broader study team was incorporated into a subsequent draft of the attributes, Luganda-translated text, and updated graphics. The participant-facing DCE included attributes and levels described in words and images purchased from image galleries or obtained freely from online sources.

**Figure 1:**
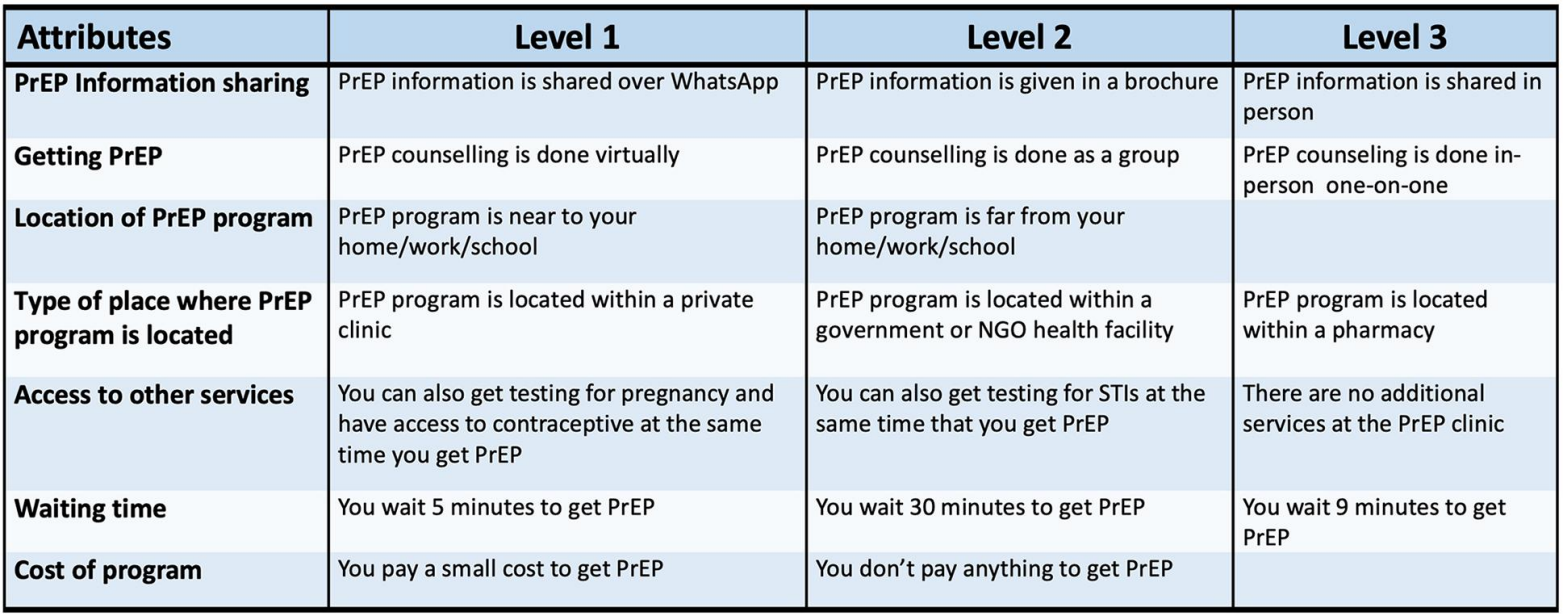
Discrete choice experiment attributes and levels.

Final attributes for the DCE included: (1) how AGYW would like to have PrEP information shared with them; (2) how PrEP counselling is delivered; (3) location of the PrEP program; (4) type of place where PrEP program is located; (5) whether other services are available at the program site; (6) how long they would be willing to wait to access PrEP and; (7) and the cost associated with the PrEP program. Piloting of the DCE was conducted prior to implementation, and the tool was revised a final time to ensure clarity. The attributes and levels represented in the DCE are shown in Figure 1.

### DCE procedures

Prior to the DCE, participants engaged in a structured, interviewer-administered survey to obtain basic demographics, relationship status, current sexual behaviour, PrEP experience, and HIV risk factors. Following the survey, a paper-based practice DCE was introduced to each participant, which consisted of two opportunities to choose their preference for local food provision. These data were used solely to familiarise participants with the DCE process and were not analysed*.* Subsequently, the DCE was administered on a password protected electronic tablet in Luganda (local language) and English. Two female and two male trained research assistants were available to administer the DCE or to assist participants in self-completing the DCE based on their preference and comfort level with the tablet.

Each participant responded to nine hypothetical PrEP program choice sets, which asked her to choose between two different PrEP program scenarios. We excluded an option to choose “neither” of the PrEP program scenarios presented. To create a full set of hypothetical scenarios, a fractional factorial choice matrix was constructed for seven attributes, each with 2–3 levels. A D-efficient non-orthogonal design was used to generate the choice matrix, aiming to maximize statistical efficiency. Choice sets were then randomly assigned using the balanced overlap method. The DCE was designed and administered using Sawtooth Software Lighthouse Studio 9.15.0 (Provo, UT, USA). All data were quality-checked weekly to ensure data completeness.

### Ethics

All participants provided written informed consent in English or Luganda. Participants ages 16–17 years were considered emancipated minors and provided written consent. The study protocol received ethical approval from the University of Alabama at Birmingham Institutional Review Board (IRB-300011438), Infectious Diseases Institute Research Ethics Committee (IDI-REC-2023-61) and the Uganda National Council for Science and Technology (HS3665ES).

### Statistical methods

Summary statistics were calculated to describe participant sociodemographic characteristics and PrEP experience. Responses to the discrete choice experiment were analysed using multinomial logit modeling. Sub-group analyses were performed using stratified modelling for participants with a) any previous PrEP use or no previous PrEP use and b) less than three partners in the prior 30 days or more than three partners in the prior 30 days. Importance scores and preference weights were reported. All analyses were conducted using SAS 9.4 (SAS Institute Inc., Cary, NC, USA) and Sawtooth Software Lighthouse Studio 9.15.0.

## Results

Of the 376 screened participants, 300 AGYW met the eligibility criteria, provided consent, and completed the DCE. All 76 AGYW who screened out were ineligible due to age. The median age was 21 years [interquartile range (IQR): 20–23]. Mobile phone ownership was very common (92%), including 68.5% who owned a smartphone (Table 1). Among AGYW, 85% reported being sexually active, and the median number of partners in the past 30 days was 3 (IQR: 1–15). Additionally, 29% of AGYW reported ever being diagnosed with a sexually transmitted infection (STI). A total of 38.3% of participants reported having ever used oral PrEP; of those, 42.6% (16.3% of total cohort) were currently using PrEP at the time of the DCE.

**Table 1:** Characteristics of study participants.

Characteristic	DCE participants
	*N* = 300
	N (%) or Median (IQR)
Age	21.0 (20–23)
Country of birth	
Uganda	288 (96.0%)
Other	12 (4.0%)
Highest level of education	
Primary school	85 (28.3%)
Secondary school	157 (52.3%)
Technical college or university	58 (19.3%)
Owns a mobile phone	276 (92.0%)
Owns a smartphone	189 (68.5%)
Sexually active	255 (85.0%)
Sexual partner gender preference	
Men only	253 (99.2%)
Both men and women	2 (0.8%)
Had vaginal intercourse within the past month	226 (88.6%)
Number of times had sex in the last 30 days	7 (3–24)
Sexual partners in the last 30 days	3 (1–15)
Type of current sexual partners, *not mutually exclusive*	
Regular partner	158 (69.9%)
Casual partner	123 (54.4%)
Other	2 (0.9%)
Relationship status	
Married	37 (12.3%)
Primary partner, but not married	167 (55.7%)
Casual partner	33 (11.0%)
Not in a relationship	61 (20.3%)
Other	2 (0.7%)
Uses condoms some of the time or more frequently	182 (60.7%)
Ever used PrEP	115 (38.3%)
Currently using PrEP	49 (42.6%)
Where PrEP is accessed, among those currently using PrEP	
Research study	12 (24.5%)
Private clinic	2 (4.1%)
Government clinic or hospital	32 (65.3%)
Other	3 (6.1%)
Currently using contraception	217 (72.3%)
Ever diagnosed with an STI	88 (29.3%)

**Figure 2:**
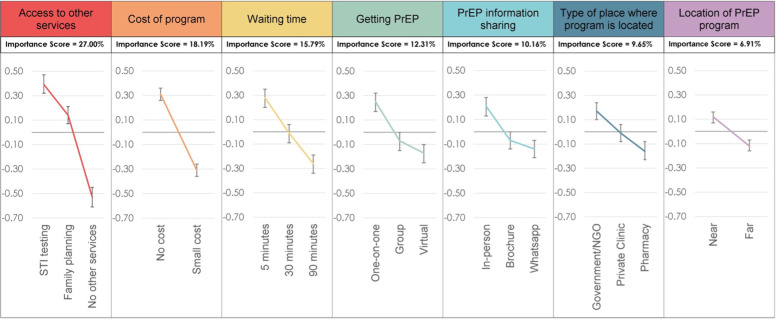
Overall DCE results for each attribute and level.

The “access to other services” attribute exerted the most influence on PrEP program choice (importance score: 27.0%) (Figure 2). Participants had a strong preference for STI testing services [preference weight [PW]: 0.39, 95% confidence interval [CI]: 0.32, 0.47] and a moderate preference for family planning services (PW: 0.14, 95% CI: 0.07, 0.21). The “cost of program” attribute was the second most influential program attribute (importance score, 18.2%) with participants preferring no cost (PW: 0.31, 95% CI: 0.26, 0.36) vs. paying a small cost for PrEP (PW: −0.31, 95% CI: −0.36, −0.26).

Other attributes had lower importance and were not very influential on choice, including “wait time” (importance score: 15.8%), “getting PrEP” (referring to how PrEP counselling is delivered) (importance score: 12.3%), “type of place where the PrEP program is offered” (importance score: 9.7%), method of “PrEP information sharing” (10.2%), and “location of PrEP program” (denoting nearness of the program to home, work or school) (6.9%). In subgroup analyses, attribute importance and preferences were similar for a) PrEP-experienced and PrEP-naïve participants and b) participants with fewer than three and three or more partners in the past 30 days.

## Discussion

The findings from this DCE among 300 Ugandan AGYW highlight the importance of bundling PrEP delivery alongside other salient health services, such as family planning and STI treatment, with lesser emphasis on factors such as mode of counselling and proximity to services. In this study, 85% of AGYW reported being sexually active, and participants' strong preference for STI testing services suggests that they view these services as a critical component of their overall sexual and reproductive health needs. This is especially important in Uganda, where access to regular STI testing is still limited (16), showing a gap between the services available and what AGYW need. Numerous studies, including systematic reviews, have documented a high prevalence of STIs among AGYW in sub-Saharan Africa, highlighting the need to integrate STI prevention into broader HIV prevention strategies (17).

Additionally, the preference for integrating PrEP with family planning services among AGYW in our DCE suggests that PrEP programs designed to include broader reproductive health support may enhance PrEP acceptability and uptake, aligning with research that supports the view that offering PrEP alongside other essential health services improves both uptake and retention (18). This is consistent with the Uganda Ministry of Health's current plan to integrate reproductive health services at public health clinics and move away from stand-alone HIV/PrEP services with dedicated staffing. The goal is to create a more unified and sustainable health system by integrating PrEP into routine service delivery (19). As Uganda launches delivery of injectable PrEP (cabotegravir recently became available and lenacapavir is anticipated to become available in 2026), there is an opportunity to integrate PrEP with family planning and streamline clinic visits by aligning dosing schedules and other delivery components (e.g., providing injections for family planning and PrEP on the same day when possible). In addition, novel products, such as the dual prevention pill, may be perfectly suited to this population group since it provides oral family planning and PrEP in one pill and multiple months of pills can be provided at once (20, 21).

Cost emerged as the second most influential DCE attribute, with participants expressing a clear preference for free PrEP services. This finding aligns with literature demonstrating that financial barriers significantly impact healthcare access, particularly for young people from resource-limited settings (22). A recent review of PrEP uptake among AGYW in Eastern, Southern, and Western Africa identified cost as a significant barrier deterring AGYW in sub-Saharan Africa from accessing PrEP (23). Recent shifts in international donor funding priorities and the introduction of more expensive PrEP products have raised concerns about the sustainability of free PrEP for AGYW in sub-Saharan Africa (24), and national-level directives have called for formerly dedicated PrEP services to be integrated with other clinic services in Uganda. As such, future programs will need to keep costs low to initiate PrEP use and support uptake. Encouragingly, our DCE finding that bundling PrEP with other health services could improve uptake and retention offers support for the Uganda Ministry of Health's recent directive to integrate PrEP into routine clinic services (19, 25). With the dramatic reduction in USAID funding and subsequent reduction of HIV prevention and treatment staff, the Ministry aims to alter the situation with parallel systems of HIV-specific vs. general health services (26). This integrated approach not only reflects health system realities but also aligns with the preferences of AGYW as supported by our DCE results.

While attributes such as wait time, service location, and information-sharing methods were ranked as lower priorities in this DCE, they nonetheless play a meaningful role in shaping the accessibility and acceptability of PrEP services among AGYW. Prior research supports that long wait times at health facilities can discourage young women from initiating or continuing PrEP (27). Similarly, the physical location of services, whether in youth-friendly clinics, community-based sites, or through mobile outreach, can significantly impact uptake by influencing convenience, perceived privacy, and safety (28). These findings show that even less prioritized service features should be important considerations when designing PrEP delivery models that meet the needs of AGYW. Programs can choose to adapt delivery approaches that work best for specific contexts and the preferences of AGYW as long as the PrEP services are bundled with other essential health services (29).

The relatively lower importance placed on being close to a clinic offering PrEP suggests that AGYW might be open to traveling for PrEP access if the service provision is satisfactory, such as being able to access adjacent health services or experiencing shorter wait times. However, this finding may reflect the setting, as the young women in this study live in an area with more health services and easier transport than many other parts of the country. A qualitative study in Kenya explored young women's experiences accessing PrEP at the same pharmacies where they obtained contraception. Participants disclosed that spending less time at the pharmacy encouraged them to return for refills, unlike at health facilities where long wait times were discouraged, ultimately impacting PrEP adherence (30). Additionally, pharmacies may consider delivery options for PrEP products that do not need clinician delivery (e.g., oral products) as they provide faster and more discrete services (31).

The similarity in attribute preferences between PrEP-experienced and PrEP-naïve participants suggests that programs can benefit both groups equally. The consistency of preferences across participants with different levels of sexual activity indicates that targeted PrEP promotion strategies may not need to vary significantly based on number of sexual partners or whether clients are engaged in transactional sex. Our DCE results suggest that one streamlined PrEP-offer approach can meet the needs of many young women, regardless of their background or experiences, resulting in establishing cost-efficient programs in locations where funding and staff are limited.

The strengths of our study include the inclusion of a large number of AGYW who were recruited from a diverse set of service organizations and research programs and our methods that incorporated DCE practice questions and opportunity for participants to become acquainted with the question format before responding. We were, however, limited in the number of attributes and levels that we could include in the DCE as we wanted to reduce the amount of time for participant engagement and maintain high study rigor. Another limitation is that our DCE excluded an option for participants to tell us that “neither option” would be preferable to the ones offered, providing us with clear data on preferences but no data on absolute preferences. Since our participants were from the Kampala, Uganda area, an urban setting, and seeking health care, results may not be generalizable to other settings, and the cross-sectional nature of the DCE provides information only about this moment in time.

## Conclusions

In this DCE, we found a strong preference for PrEP delivery to be integrated within family planning, reproductive health services and STI testing/treatment services. The provision of PrEP as free-of-charge also influenced participants' choice for PrEP programming, suggesting the need for comprehensive, affordable, multi-service healthcare models. Ensuring that PrEP is provided at no cost will not only support initiation but encourage sustained use—both key factors as AGYW experience dynamic degrees of HIV risk and navigate stigma associated with accessing sexual and reproductive health services. While factors such as wait times and service location were less influential, addressing these considerations has the potential to improve overall user experience and retention. As PrEP programs strive to include new products, including injectable cabotegravir and lenacapavir, it is crucial to use insights from AGYW to shape sexual and reproductive health services that meet their needs and support both PrEP initiation and retention and that we leverage implementation science research opportunities to test different delivery strategies. At the same time, new PrEP products are being rolled out amid significant shifts in global funding and policy landscape. How PrEP is delivered in Uganda is now part of a tidal shift from stand-alone HIV and PrEP services toward full integration into routine care. Presenting and interpreting these DCE findings among this monumental transformation is vital for the future of PrEP use and the programs that support sustainability and uptake.

## Data Availability

Data are available upon request to the corresponding author and after discussion about the research question to be answered.
